# Plasticity in metabolism of maternal androgens in avian embryos

**DOI:** 10.1038/s41598-023-35340-z

**Published:** 2023-05-18

**Authors:** Yuqi Wang, Bernd Riedstra, Bonnie de Vries, Martijn van Faassen, Alle Pranger, Ido Kema, Ton Groothuis

**Affiliations:** 1 Groningen Institute for Evolutionary Life Sciences, University of Groningen, Groningen, The Netherlands; 2 University Medical Center Groningen, University of Groningen, Groningen, The Netherlands

**Keywords:** Developmental biology, Ecology, Evolution, Physiology

## Abstract

Mothers can influence offspring phenotypes by transferring non-genetic information to the young, which provides them with a flexible tool to adjust the developmental trajectory of the young in fluctuating environments. Mothers can differentially deposit their resources in the same reproductive attempt in relation to the offspring position in the sibling hierarchy. However, whether embryos from different positions can be plastic in their response to the maternal signals, potentially leading to a mother–offspring conflict, is yet unclear. We used Rock pigeons (*Columba livia*), that lay two egg clutches where maternal androgen levels in second laid eggs at oviposition are higher than in first laid eggs, and investigated the plasticity of embryonic metabolism of maternal androgens. We experimentally elevated androstenedione and testosterone levels in first eggs to that present in second eggs and measured the change in androgen levels and its main metabolites (etiocholanolone and conjugated testosterone) after 3.5 days of incubation. We found that eggs with increased androgens show a different degree of androgen metabolism depending either on the egg laying sequence or initial androgen levels or both. Our findings indicate that embryos have certain plasticity in response to maternal androgen levels depending on maternal signals.

## Introduction

Mothers can affect the development of their offspring not only by transferring information via their genes, but also by transferring non-genetic signals such as nutrients, immune factors, and hormones. Such transfer can depend on the maternal environment which provides the mother with a tool to adjust the developmental trajectory and final phenotype of their offspring to environmental fluctuations, contributing to fitness by so called adaptive anticipatory maternal effects^1^. Over the past decades it has become increasingly clear that such effects occur already prenatally by exposing embryos or provisioning eggs differentially with both resources and signals such as nutrients, and hormones (e.g. Insects^2,3^, reptiles^4–6^, birds^7–9^ and mammals^10,11^). A classic example, in birds, is the maternal transfer of androgens that vary (often increase) with the egg laying sequence in the same reproductive attempt^7^. Such variation would boost the development of later-laid eggs at the cost of earlier-laid eggs under good food conditions, yet reduce the early survival of the later-laid eggs under poor food conditions which leads to brood size reduction^12,13^.

So far, extensive studies have accumulated that enable us to understand the role of the mother and the effects of her hormones on the offspring in the post-natal stage. At the same time, increasing evidence indicates that the embryo may not simply be a passive recipient of maternal signals^14,15^. A well-known example is that of the human fetus that can increase its nutritional availability by secreting placental hormones that affect its mother’s physiology, leading to an increase of maternal blood glucose and blood pressure^16^. Such cases have been interpreted as the early expression of a mother–offspring conflict^17^. This raises the question to what extent the embryo is plastic in its response depending on contextual cues as predicted by theory^7,14,15,18^.

Over years, descriptive and experimental studies in oviparous species have found hormones of maternal origin in substantial amounts in the egg yolk of these species^7,19–23^ having a wide array of beneficial as well as detrimental effects on offspring development^7,24,25^. Several studies have now shown an active role of embryos in converting the maternal hormones to other hormones^6,26–29^. Such metabolism occurs already in the very early days of development which can have important biological effects and either function directly, such as etiocholanolone functioning as neurosteroids^30^ or indirectly, such as inactive conjugates being converted back to the active free form later during embryonic development^6^. However, whether or not metabolism of maternal substances is an active plastic response strategy by embryos depending on contextual cues, potentially to tailor phenotypic development towards their own personal fitness optimum, remains unclear.

Evidence for such plasticity was reported recently by Kumar et al.^29^ who observed that Rock Pigeon embryos (*Columba livia*) differ in their conversion rate of maternal androgens according to their position in the laying sequence, and enzymes needed for the conversion were produced by the embryos^31^. This species produces two-egg clutches where second-laid eggs contain much higher maternal androgen levels than first-laid eggs but both declined to similarly low levels within the first few days of incubation. As a consequence, the second-laid eggs end up with higher levels of metabolites, such as etiocholanolone and conjugated steroids. There are two possible mechanistic explanations for these results: either embryos increase hormone conversion rate with increasing initial androgen exposure, or embryos from different laying sequence metabolize maternal androgens according to maternal cues that indicate the embryo’s position in laying sequence. Hereafter, these two scenarios will be referred to as the initial level dependent hypothesis and the laying sequence dependent hypothesis. For both hypotheses there is some supporting functional evidence. A study in the same species indicated that increased yolk androgens of the first egg increase chick’s body mass and therefore its competitiveness within the brood^32^. A few other studies indicated that the effect of testosterone treatment differ between chicks hatched from different positions in the laying sequence (Zebra finche ﻿*Taeniopygia guttata*^9^; Black-headed gull *﻿Larus ridibundus*^12^). Moreover, it has been shown that androgen receptors are present in the embryo before they produce their own androgens, which has been interpreted as a strong argument for adaptation to maternal androgens, transforming the signal into functional consequences^33^. Evidence for either hypotheses would reveal which maternal cue(s) the embryos are following in metabolizing maternal androgens and further indicate embryonic plasticity in response to maternal signals that potentially mediates maternal-offspring conflict under different context.

**Figure 1:**
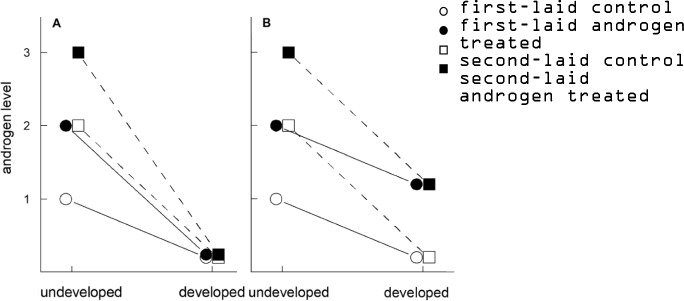
Conceptualization of the two hypotheses. First-laid eggs (open circles) have lower levels of maternally derived androgens and have lower rates of androgen conversion than second-laid eggs (open squares) but end up with similarly low levels during 3.5 days of development. (**A**) The initial level dependent hypothesis—if the difference in conversion rate is caused by the initial level of androgens deposited by the mother in the egg, then increasing androgen levels would increase conversion rates, resulting in levels that are similar to those in control eggs after 3.5 days development. (**B**) The laying sequence dependent hypothesis—if the difference in conversion rate is due to intrinsic egg characteristics associated with laying sequence, then experimentally increasing androgen levels (closed symbols) should result in higher androgen levels in both first and second laid eggs after 3.5 days of development, compared to unmanipulated eggs.

To disentangle these two hypotheses, we experimentally elevated androgen levels (androstenedione and testosterone) in first eggs to the levels that are typical for second eggs of rock pigeons, and measured the change in androgen levels and its main metabolites (etiocholanolone and conjugated testosterone) after 3.5 days of incubation. To complement the study, we also injected second eggs with the same dose of androgens as in the first eggs. We expected that, if the initial level hypothesis is correct, first-laid androgen treated eggs with would “behave” like control second-laid eggs, while second-laid androgen treated eggs show a further increase in conversion rate (see Fig. 1A). However, if the laying sequence dependent hypothesis is correct, the androgen conversion rate would dependent on the laying sequence (see Fig. 1B). In addition, we measured several metabolites (conjugated testosterone, conjugated etiocholanolone, and etiocholanolone) to investigate whether the embryos produce these in different percentages to the initial androgens they possessed, which would be further evidence for embryonic plasticity.

## Methods

### Animal housing

﻿Embryos were obtained from eggs of forty pairs of Rock pigeons that were housed in an outdoor aviary (45 m length × 9.6 m width × 3.75 m height) under ambient light and temperature conditions at the University of Groningen, the Netherlands, with ad libitum access to fresh water and food. Food contained a mixture of commercial pigeon seeds (Kasper 6721 and Kasper 6712), P40 vitamin supplement (Kasper P40), and grit. Fifty nest boxes (dimensions: 60 cm × 50 cm × 36 cm) were placed on one of the long sides of the aviary in 1-m intervals at a height of 1.5 m. Breeding bowls were placed in the nest boxes and nesting materials were provided in the aviary. Food and water availability, nest building, and egg laying were checked daily. All animal procedures were approved by the animal welfare committee of the University of Groningen and carried out under the guidelines of that committee.

### Egg collection and incubation

All nest boxes were assigned with a fixed identity and checked twice daily at 12:00 h and 19:00 h from March to June 2019 to collect freshly laid first and second eggs. At collection, each first laid egg was replaced with a dummy egg. After collecting the second egg, dummy eggs were taken away and birds were allowed to produce a second clutch. On the day of collection, eggs were marked with a unique egg ID and then transported to our inside facility. Here they were weighed to the nearest 0.1 g, and the length and width were determined to the nearest 0.1 mm using a sliding caliper whereafter they were injected with an androgen or a control solution (see below). Treatment was randomized with respect to nest and laying sequence, but on each day half of the eggs were injected with the hormone solution and the other half with the control solution. Directly after injections, eggs were placed in an incubator at 37.4 ℃, 55% relative humidity and automatic egg turning every 12 h (Ova-Easy Advance Series II Digital Cabinet Egg Incubator, Brinsea Products Inc., 704 N Dixie Ave., Titusville, FL 32796–2017 USA). After 3.5 days of incubation, at which time point a significant amount of maternal androgen metabolism has occurred, but endogenous androgen production has not yet started^24^, eggs were candled to determine developmental stage. Eggs showing no signs of development were identified as undeveloped eggs, which considered to have hormone levels representing the eggs at oviposition^29^. The remaining eggs were considered as developed eggs. All eggs were frozen at − 20 °C for later hormone analysis. In total, 10 out of 37 first laid eggs and 12 out of 40 s laid eggs in the control group showed signs of development; 11 out of 42 first laid eggs and 11 out of 38 s laid eggs in the androgen treatment group showed proper signs of development. We used all developed eggs and 10 undeveloped eggs from each treatment group for hormone analysis. Hormone treatment did not cause a significant selection effect on embryo survival (ANOVA, F = 0.02, *p* = 0.88).

### Egg injections

Directly after collection, eggs were placed horizontally on an egg holder for 5 min to allow the yolk to float to the top. Then, a cotton pad containing 75% ethanol was applied to disinfect the eggshell and a small hole was drilled in the eggshell on the top, approximately 2 mm beside the central axis, and approximately at two-thirds of the egg toward the air chamber. Then a disposable insulin syringe (U-100, 29G needle × 12.7 mm, BD Micro-Fine) was inserted through the hole with an angle of approximately 15 degrees to inject either 50 µl sesame oil (vehicle) or 50 µl sesame oil containing a mixture of 144 ng/mL testosterone (T; art.no. 86500-1g, Sigma) and 2210 ng/mL androstenedione (A4; art.no.46033-250mg, Sigma). These concentrations were based on the difference in these hormone levels between first and second laid eggs as reported by Kumar *et al.*^29^ and increase therefore the total amount of T and A4 in first laid eggs to the total amount in second laid eggs. We also increased the level of T and A4 in second laid eggs with this amount which increased T and A4 by approximately 1.5 times. This increase is within the average level plus two times the standard deviation^34^ and considered safe for embryonic development. After injection, a drop of Vetbond (3M, USA) was applied to seal the puncture in the egg shell.

### Hormone extractions and mass spectrometric analyses

Hormone extractions and analyses followed Kumar *et al.*^29^. In brief, eggs were thawed at room temperature and the entire egg was homogenized (except shells). For each egg, 300 mg of the homogenate was used for liquid chromatography combined with tandem mass spectrometry (LC–MS/MS) to determine the level of testosterone, conjugated testosterone, and androstenedione (conjugated androstenedione was excluded as a target because it lacks a free hydroxyl group in its structure and hence cannot be conjugated). Meanwhile, 600 mg of the homogenate was used for gas chromatography combined with tandem mass spectrometry (GC–MS/MS) to determine the level of etiocholanolone and conjugated etiocholanolone. Internal standard (For LC–MS/MS: 25 μL of 30 nmol/L ^13^C_3_ labelled testosterone in 50% methanol, IsoSciences; For GC–MS/MS: 100 μL: of 6.7 µmol/L ^2^H_5_ labelled etiocholanolone in 100% methanol, IsoSciences) was added to the samples, mixed thoroughly and left for one hour at room temperature for equilibration. Then each sample was extracted twice in 1 ml methanol. The supernatant was then transferred to tubes containing solid ZnCl_2_ (200 mg for LC–MS/MS; 300 mg for GC–MS/MS) for lipid precipitation. Final eluate was obtained through C18 columns (#5138775, Aurora Borealis) for LC–MS/MS or HLB cartridges (#WAT094226, Waters Chromatography BV) for GC–MS/MS. The eluate was then divided into two equal parts, proceeded with or without hydrolysis. The procedure of hydrolysis deconjugates the conjugated hormones into their free forms. The levels of conjugates were therefore calculated as difference of the parts proceeded with hydrolysis and the parts proceeded without hydrolysis. All samples were then transferred to University Medical Center Groningen for chromatographic separation and mass spectrometry. The detection limit was 0.025 nmol/L for free form testosterone, 0.01 nmol/L for free form androstenedione and 1 nmol/L for free form etiocholanolone.

### Statistical analysis

All data were analyzed with the software R (version 4.0.4), with package *lme4* for Linear Mixed Models (LMMs)^35^.

The data of hormone levels in undeveloped eggs had a skewed distribution (Shapiro test with *p* < 0.05). Therefore, we compared the hormone levels in undeveloped control and androgen treated eggs using Mann–Whitney U-test to check the effectiveness of our treatment. As expected, the levels of etiocholanolone and conjugated etiocholanolone, both generated by embryonic conversion that should be lacking in these undeveloped eggs, were under the detection limit for almost all undeveloped eggs and were excluded from these analyses. Therefore, we only analyzed T, A4 and conjugated T. We also pooled all experimental groups separately for undeveloped eggs and developed eggs and used t-tests to analysis the effects of development on the changes of each hormone.

To examine the active role of the embryo in steroid dynamics during early incubation and test our two hypotheses, we tested our expectations (Fig. 1) by means of LMMs (with normal distribution due to the Central Limits Theorem) with hormone levels in undeveloped and developed eggs as dependent variables, and development (yes or no), egg sequence (1 or 2), treatment (androgen or vehicle injection), interaction of development and egg sequence and interaction of development and treatment as predictors, female identity (which equals to nest identity as females have a very high fidelity to the nest box) as random intercept and female identity as a random coefficient for egg laying sequence. We excluded the interaction of egg sequence and treatment as a predictor in our model as it was not in our interest (i.e. not being formulated in the two hypothesis).

To test whether, despite potential differences in the rate of hormone conversion among different groups, eggs still differed in hormone levels after 3.5 days of incubation, we used Mann–Whitney U-tests (as the data displayed skewed distributions (Shapiro test with *p* < 0.05)).

To study whether embryos showed also plasticity in the proportions of the metabolites converted from the initially deposited androgens (androstenedione, testosterone and conjugated testosterone) into different percentages of metabolites, depending on the experimental group, we calculated the group-wise average quantity of each androgen (nmol/egg) of the undeveloped eggs and set the sum of them (as total androgens) to 100% (Supplementary Table 1). Then we calculated the percentages of the androgens and their metabolites of developed eggs for each group, where the difference (the decrease) in the total amount of measured hormones was assigned as unknown metabolites (Supplementary Table 2). Kendall’s coefficient of concordance was used to assess the percentage difference among groups. T-tests were used as post-hoc to compare the percentages difference of converted androgens and the metabolites between groups.

## Results

### Validation of the design in undeveloped eggs

As expected, undeveloped second-laid control eggs showed higher testosterone and androstenedione levels compared to undeveloped first-laid control eggs (Table 1). Moreover, androgen treatment effectively increased androstenedione and testosterone levels in the undeveloped first-laid eggs (Table 1) to that of second-laid control eggs, resulting in no significant differences between both (Table 1). Also, testosterone and androstenedione levels in undeveloped eggs were higher in androgen treated second-laid eggs than second-laid control eggs (Table 1).

**Table 1:** Pairwise comparisons of hormone levels between first- and second-laid, control and androgen treated among undeveloped eggs. Etiocholanolone and conjugated etiocholanolone were mostly under detection limit in undeveloped eggs and therefore excluded. W and *p*-values from Mann–Whitney U tests. 1C = first-laid control eggs, 2C = second-laid control eggs, 1A = first-laid androgen treated eggs, 2A = second-laid androgen treated eggs.

Hormone	Statistical parameters	Comparison between groups			
		1C–2C	1C–1A	1A–2C	2C–2A
Androstenedione	W-statistic	0	0	40	6
	*p* -value	** < 0.001**	** < 0.001**	0.48	**0.001**
Testosterone	W-statistic	0	0	29	0
	*p* -value	** < 0.001**	** < 0.001**	0.12	** < 0.001**
Conjugated testosterone	W-statistic	51	53	39	37
	*p* -value	0.66	0.31	0.65	0.53

Significant are in value [bold].

### Differentiating between the two hypotheses

**Figure 2:**
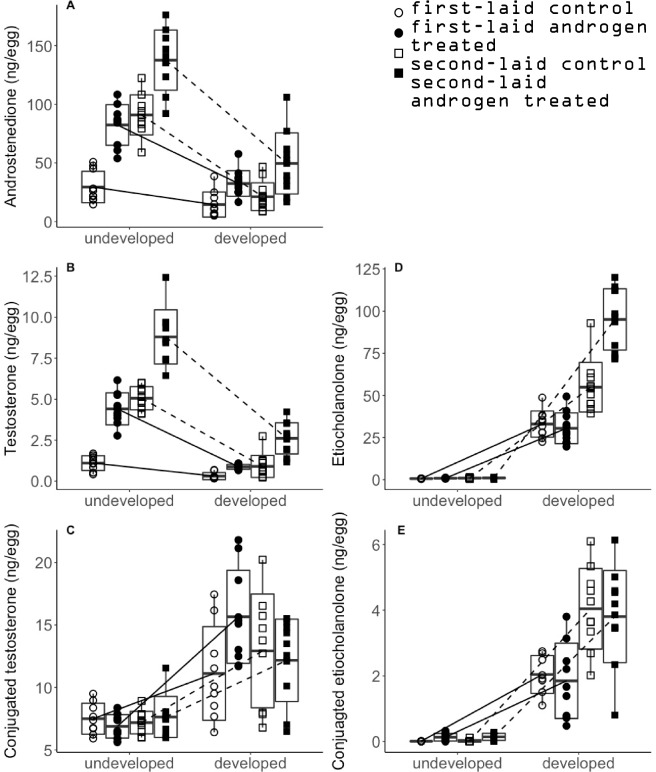
Levels of hormones in undeveloped and developed eggs, both incubated for 3.5 days. Panel A–E depict the androstenedione, testosterone, etiocholanolone, conjugated testosterone and conjugated etiocholanolone levels in whole eggs. Middle bar, hinges and whiskers of the box represent mean values, standard deviation and min–max values respectively.

We replicated the findings of Kumar et al.’s^29^ because both androstenedione and testosterone levels strongly decreased during early development while the three metabolites clearly increased (t-tests, all *p* < 0.001, also see Fig. 2). Additionally, there were significant interactions between development and treatment on post-incubation levels of both androstenedione and testosterone (Table 3): supporting the initial level dependent hypothesis since eggs with increased androgens metabolized significantly more of both hormones than control eggs (Fig. 2A,B). Yet despite their more rapid metabolism, eggs with increased androstenedione and testosterone levels still ended up with higher levels of these androgens than their control groups (Table 2). Moreover, for both testosterone and androstenedione, the interaction between development and laying sequence was also significant (Table 3): supporting the laying sequence dependent hypothesis since second-laid eggs metabolized more testosterone and androstenedione than first-laid eggs irrespectively of their initial hormone concentrations (Fig. 2B). Yet second laid eggs still ended up with higher testosterone but not androstenedione levels (Table 2). Finally, androgen treated first-laid eggs ended up with higher testosterone levels but equal androstenedione levels compared to second-laid control eggs (Table 2).

**Table 2:** Pairwise comparisons of hormone levels between first- and second-laid, control and androgen treated among developed eggs.

Hormone	Statistical parameters	Comparison between groups			
		1C–2C	1C–1A	1A–2C	2C–2A
Androstenedione	W-statistic	33	12	105	18
	*p* -value	0.08	**0.002**	**0.02**	**0.002**
Testosterone	W-statistic	15	3	76	8
	*p* -value	**0.004**	** < 0.001**	0.31	** < 0.001**
Conjugated testosterone	W-statistic	31	17	57	53
	*p* -value	0.44	**0.02**	0.36	0.55
Etiocholanolone	W-statistic	4	67	5	4
	*p* -value	** < 0.001**	0.43	** < 0.001**	** < 0.001**
Conjugated etiocholanolone	W-statistic	6	43	9	59
	*p* -value	**0.001**	0.54	**0.002**	0.81

Significant are in value [bold].

**Table 3:** Summary of the LMM describing variation in androgen levels. Parameters are present as (estimate ± standard error) with significant terms as superscript.

	Androstenedione	Testosterone
Intercept	(30.9 ± 4.78)**	(1.10 ± 0.22)***
Development^1^	(− 19.3 ± 6.70)**	(− 1.08 ± 0.31)**
Treatment^2^	(49.9 ± 5.52)***	(3.32 ± 0.27)***
Laying sequence^3^	(58.4 ± 5.53)***	(4.16 ± 0.30)***
Development^1^ × treatment^2^	(− 26.4 ± 7.63)***	(− 2.20 ± 0.36)***
Development^1^ × laying sequence^3^	(− 46.5 ± 7.64)***	(− 2,92 ± 0.37)***

^1^Relative to undeveloped eggs.

^2^Relative to control eggs.

^3^Relative to first-laid eggs.

*** p -value < 0.001; ^ns^ *p* -value > 0.05.

### Plasticity in the androgen metabolism

**Figure 3:**
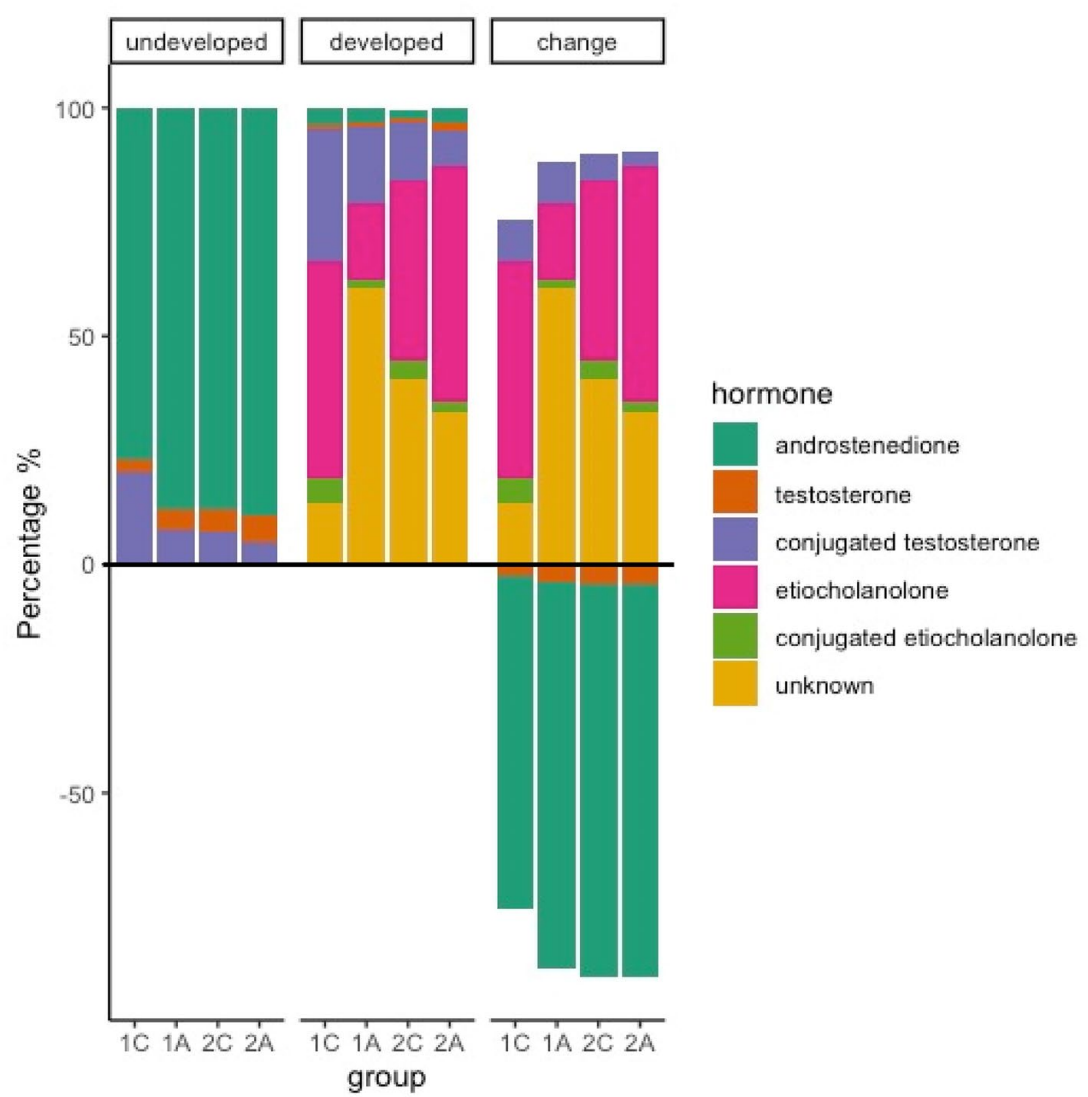
Percentages of hormones to total androgens present in undeveloped eggs. The facets show the percentages in undeveloped, developed eggs, and the percentage change between developed and undeveloped eggs (negative values mean decrease and positive values mean increase). 1C = first-laid control eggs, 2C = second-laid control eggs, 1A = first-laid androgen treated eggs, 2A = second-laid androgen treated eggs.

The percentages of androgens and their metabolites in developed eggs were different among groups (Kendall’s W = 0.91, *p* = 0.003 Fig. 3). Post-hoc analyses showed that the percentage of converted androgens was higher for second-laid control eggs than first-laid control eggs and it was increased by androgen treatment in first-laid eggs but, interestingly, not in second-laid eggs (Supplementary Fig. 1A). For conjugated testosterone, its percentage was lower for second-laid control eggs than first-laid control eggs while androgen treatment decreased the percentage for both first- and second-laid eggs (Supplementary Fig. 1B). For etiocholanolone, androgen treatment decreased its percentage in first-laid eggs but not second-laid eggs (Supplementary Fig. 1C). Finally, first-laid control eggs had lower percentage of unknown metabolites than second-laid control eggs, while androgen treatment increased the percentage of the former but not in the latter (Supplementary Fig. 1E).

## Discussion

In this study, we provide experimental evidence for an active, context-dependent role of the embryo very early in development in determining the concentration of maternally-derived androgens and their metabolites to which they will be exposed to. We showed that the conversion rate of both androstenedione and testosterone during development was depending on the initial levels of the hormones in the eggs, such that after 3.5 days of incubation the initial differences between the eggs was strongly reduced. In addition, the decrease in androstenedione and testosterone was also depending on the position of the embryo in the laying sequence. Furthermore, the different proportions of the other hormones in which androstenedione and testosterone and were converted to differed according to both the initial androgen levels and the laying sequence. These results not only demonstrate the importance of investigating the role of the young embryo for understanding hormone-mediated maternal effects, but also indicate an active role of the embryo in influencing the scope for biological effects of maternal hormones on itself. This may provide the possibility for a role of the embryo in a potential mother–offspring conflict but that requires much more testing.

**Figure 4:**
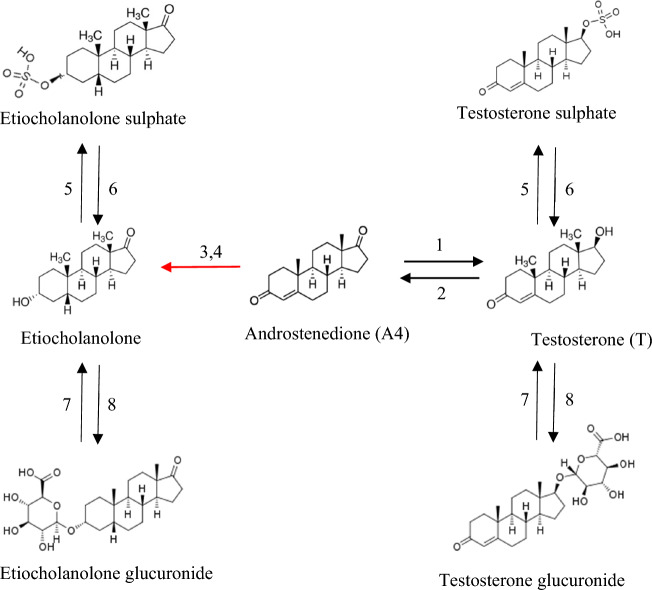
The hormone metabolic pathways studied in this paper. The arrows indicate the direction of conversion between two hormones and the numbers represent the enzymes needed for the conversion. 1 = HSD17B3, 2 = HSD17B2, 3 = 5β-reductase, 4 = 3α-HSD, 5 = sulfotransferase, 6 = sulfatase, 7 = glucuronosyltransferase, 8 = glucuronidase. Etiocholanolone sulphate and etiocholanolone glucuronide are both conjugated etiocholanolone. Similarly, testosterone sulphate and testosterone glucuronide are both conjugated testosterone.

The hormones we measure showed different dynamics during development. This may be explained by the fact that the enzymes involved in different hormone conversions are partially different (Fig. 4). Androstenedione needs 17β-HSD to be converted into (HSD17B3) or back from (HSD17B2) testosterone^36^. It needs 5β-reductase following 3β-HSD to be converted into etiocholanolone, while testosterone needs 5β-reductase, 3α-HSD following 17β-HSD to be converted into etiocholanolone^37^. It is well known that the relevant enzymes for these conversions can be expressed in chicken embryos as young as 2-days^24,38^ and hormones such as testosterone can upregulate enzyme activity relevant for its own conversion^39,40^, which may explain the partial support for the initial level hypothesis. Meanwhile, enzymes such as sulfotransferase/glucuronosyltransferase and sulfatase/glucuronidase are responsible for hormone conjugation and deconjugation during embryonic development^41^. The dynamics of the metabolites we measured, however, cannot be explained by the dynamics of their upstream androgens (i.e. androstenedione and testosterone) as the increase in the former was less than the decrease in the latter. Into what compounds the remaining androgens are converted remains to be explored.

The different percentages of androgens being metabolized and the different percentages of the metabolites among groups showed that embryos have plasticity in metabolizing maternal androgens. Interestingly, in the first- but not second-laid eggs, the percentages of androgens being metabolized and their unknown metabolites increased while the percentage of etiocholanolone decreased by androgen treatment (Supplementary Table 2, Supplementary Fig. 1A,C), which further indicates that embryos from different positions in the laying sequence may have different strategies in metabolizing maternal androgen. For the conjugates, androgen treatment decreased the percentages of conjugated testosterone and conjugated etiocholanolone in both first- and second-laid eggs, yet the percentage (as well as the absolute level) of conjugated testosterone was much higher than conjugated etiocholanolone (Supplementary Fig. 1B,D). A possible explanation for this distinctive difference between the conjugates is that, unlike conjugated etiocholanolone which cannot be converted back into testosterone, conjugated testosterone can be converted back into free testosterone and become involved in further androgen metabolism^29,41^. Therefore, embryos may have a larger capacity to conjugate testosterone. This is supported by the fact that in developed eggs, testosterone shows a much higher conjugated to free form ratio than etiocholanolone (approximately 10:1 against 1:20, see Fig. 2), which indicates that conjugated testosterone is more likely to act as a dynamic buffering pool of testosterone, which may not hold for etiocholanolone.

The active role of the embryo in dealing with maternal hormones may be the evolutionary outcome of a parent–offspring conflict over hormone exposure. As indicated in the introduction, allocating elevated levels of androgens by the mother to one offspring over the other can be beneficial for mother’s fitness, but detrimental to either of these offspring^12^, depending on the context such as food availability^32^. This is yet as speculation based on our data, replicating the experiment under both good and poor food conditions may therefore be relevant to explain the functional consequences of the embryonic metabolism of the maternal androgens.

Our study showed that the mechanism for context-dependent offspring response, is in place and probably available to be under selection. However, the mechanism under which the embryos could deal with maternal androgens against the interest of the mother requires more studies on two important issues. First, it requires the offspring to perceive the relevant context that is provided against the interest of the mother. For the laying order dependent effect, this may be changes in egg composition over the laying sequence that the mother cannot avoid because of decreasing resources during egg laying, as in several other avian species the eggs at different laying positions differ in many aspects, including yolk and albumin mass, and the amounts of antibodies, carotenoids, and vitamins^42–44^. For other contextual cues such as food availability it might be the amount of nutrients in the egg. Another possible environmental cue might be a difference in incubation temperature. Often, before clutch completion, parents start to partially incubate the earlier laid eggs to keep them viable before full incubation sets in^45^. However, in our experiment all eggs experienced the same incubation pattern in the incubator, so this factor can be ruled out for explaining our results.

For the initial level dependent effect, the data suggest that despite increased allocation of maternal androgens the embryos quickly convert them into metabolites. This raises a second important issue: we need to know more of the biological function of these metabolites. It has been suggested that the conjugated less polar forms of the free androgens are needed for taking up the free polar forms from the yolk environment into the watery embryonic circulation, and that they then become de-conjugated in the target tissues of the embryo^46,47^. Also, etiocholanolone can have effects on erythropoiesis which could in turn promote the development of the embryo^28,48–50^. Moreover, a recent review indicated that etiocholanolone can act as a neurosteroid^30^. To what extent the metabolites are biological active, and to what extent they differ in their effects affecting the final phenotype needs further study. Nevertheless, despite the increased rate of conversion of maternal hormones in second eggs or hormone treated eggs, in several cases the eggs still had somewhat higher levels of these hormones after 3.5 days of incubation, although the initial difference between the eggs before incubation was much larger. This may suggest that in the course of evolution mother and offspring reached a compromise, explaining why mothers still allocate higher levels of androgens to second eggs relative to first eggs and this difference is completely diminished by the embryo. In any case the strong early metabolism indicates that the high levels of androgens in freshly laid eggs do not function as a source for gradual uptake over the entire incubation period^45,47,51^.

In conclusion, this study suggests that developing embryos might be capable of modulating maternal androgens in a context-dependent way, already early in development. In addition to providing fundamental insights into hormone-mediated maternal effects, our findings could explain the inconsistencies of *in ovo* androgen injection studies^52^, as results may differ according to egg sequence and potentially other contextual factors influencing egg composition and or incubation patterns. It shows an intriguing new layer of complexity in hormone mediated maternal effects and highlights the importance of understanding the extent to which the embryos could regulate and optimize the maternal input, perhaps independently from the maternal interest, although that requires much further testing.

## Supplementary Information

Supplementary Information.

## Data Availability

Data are available as electronic supplementary material.
