# Supplementary material for: Plasticity in metabolism of maternal androgens in avian embryos
Source: Sci Rep. 2023 May 18;13:8083. doi: 10.1038/s41598-023-35340-z (PMC10195863; doi:10.1038/s41598-023-35340-z)

Supplementary

To assess plasticity in which part of the original androgens was converted into which metabolites we calculated the average group-wise quantity of androstenedione, testosterone and conjugated testosterone (nmol/egg) for the undeveloped eggs and set that to 100% (Supplementary Table 1). Then we calculated the percentage of the androgens being metabolized (the decreased of androstenedione and testosterone) and the percentage of the metabolites for each developed eggs (Supplementary Figure 1), where the difference between the decrease in the total amount of androstenedione and testosterone over incubation time and the increase in their metabolites (so the part of the decrease in the two androgens which was not accounted for by the metabolites we measured) was assigned the category unknown (Supplementary Table 2).

Supplementary Table 1

The group-wise average quantity of each hormone (nmol/egg) and their percentages to the total amount of androgens for undeveloped eggs. The total androgens were the total quantity of testosterone, androstenedione and conjugated testosterone in undeveloped eggs which we considered as the androgens the embryos initially possessed. Etiocholanolone and its conjugates were considered as non-existing in undeveloped eggs and therefore excluded in the table. 1C = first-laid control eggs, 2C = second-laid control eggs, 1A = first-laid androgen treated eggs, 2A = second-laid androgen treated eggs.

	testosterone	androstenedione	conjugated testosterone	Total androgens
1C	0,004 (2,91%)	0,103 (76,9%)	0,027 (20,1%)	0,133 (100%)
1A	0,015 (4,68%)	0,288 (88,0%)	0,024 (7,29%)	0,327 (100%)
2C	0,018 (4,87%)	0,318 (88,2%)	0,025 (6,90%)	0,360 (100%)
2A	0,031 (5,67%)	0,481 (89,4%)	0,026 (4,92%)	0,538 (100%)

Supplementary Table 2

The group-wise average quantity of each hormone (nmol/egg) and their percentages to the total amount of androgens for developed eggs.

	testosterone	androstenedione	conjugated testosterone	etiocholanolone	conjugated etiocholanolone	unknown metabolites
1C	0,001 (0,79%)	0,005 (3,79%)	0,039 (28,9%)	0,064 (47,6%)	0,007 (5,27%)	0,018 (13,6%)
1A	0,003 (0,93%)	0,011 (3,48%)	0,054 (16,7%)	0,055 (16,9%)	0,006 (1,70%)	0,198 (60,4%)
2C	0,003 (0,87%)	0,007 (2,06%)	0,045 (12,4%)	0,143 (39,8%)	0,014 (3,86%)	0,147 (40,9%)
2A	0,009 (1,68%)	0,017 (3,21%)	0,042 (7,83%)	0,277 (51,5%)	0,013 (2,43%)	0,179 (33,4%)

Supplementary Table 3

Marginal r-squared from GLMMs (with Gamma distribution) describing variation in androgen levels.

GLMM model	Marginal r-squared
androstenedione	0.74
testosterone	0.68

Supplementary Figure 1

Percentage of metabolized androgens and their metabolites in developed eggs. P-values from t-tests are shown. 1C = first-laid control eggs, 2C = second-laid control eggs, 1A = first-laid androgen treated eggs, 2A = second-laid androgen treated eggs.

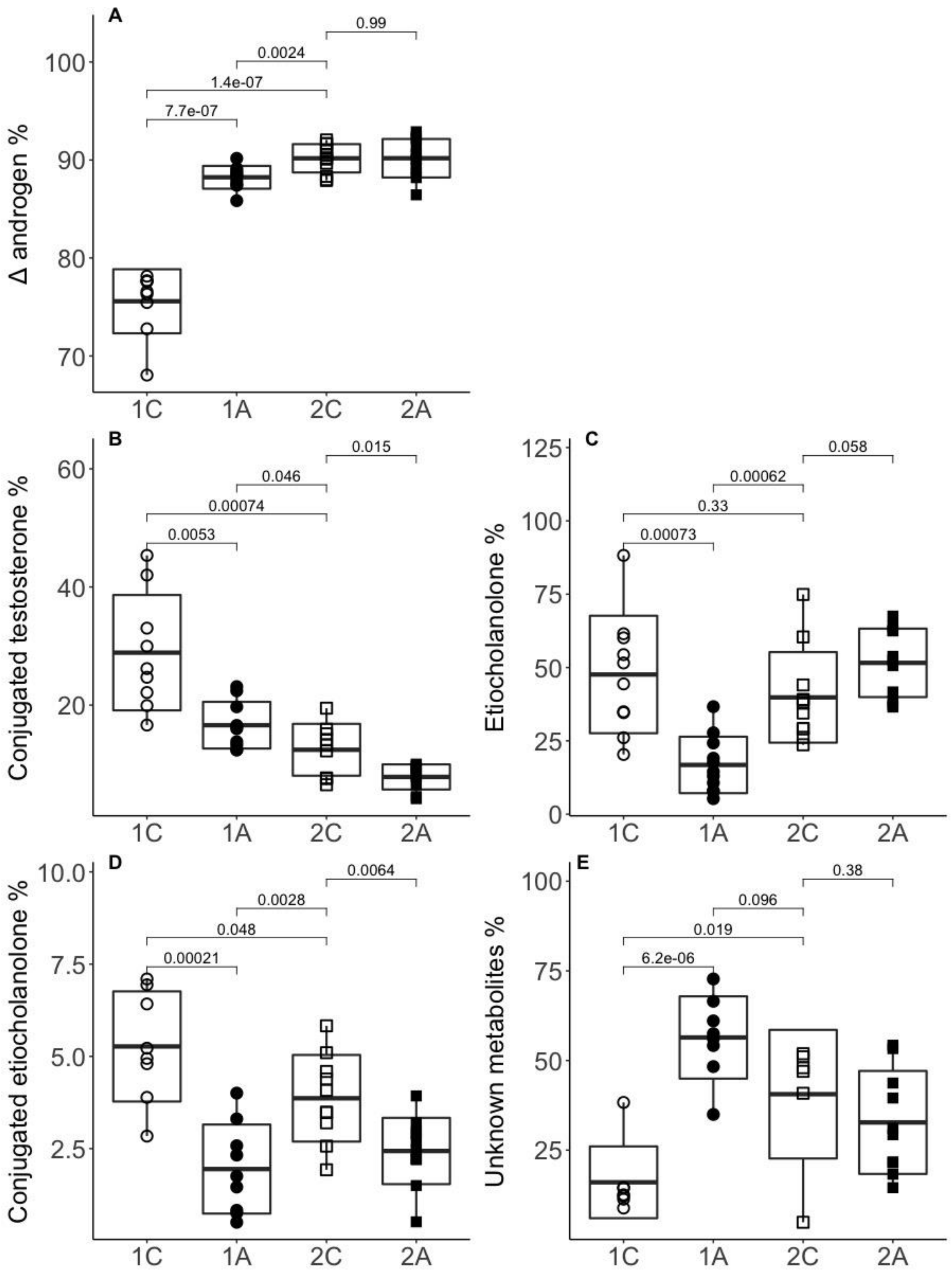

Supplement: Supplementary file 1 — Supplementary Information. [file 41598_2023_35340_MOESM1_ESM.pdf]
